# The Voltage-Gated Sodium Channel in *Drosophila*, Para, Localizes to Dendrites As Well As Axons in Mechanosensitive Chordotonal Neurons

**DOI:** 10.1523/ENEURO.0105-23.2023

**Published:** 2023-06-27

**Authors:** Thomas A. Ravenscroft, Ashleigh Jacobs, Mingxue Gu, Daniel F. Eberl, Hugo J. Bellen

**Affiliations:** 1 Department of Human and Molecular Genetics, Baylor College of Medicine, Houston, TX 77030; 2 Neurological Research Institute, Texas Children’s Hospital, Houston, TX 77030; 3 Department of Biology, University of Iowa, Iowa City, IA 52242; 4 Department of Neuroscience, Baylor College of Medicine, Houston, TX 77030

**Keywords:** AIS, channel, mechanosensors, para, PNS, sodium

## Abstract

The fruit fly *Drosophila melanogaster* has provided important insights into how sensory information is transduced by transient receptor potential (TRP) channels in the peripheral nervous system (PNS). However, TRP channels alone have not been able to completely model mechanosensitive transduction in mechanoreceptive chordotonal neurons (CNs). Here, we show that, in addition to TRP channels, the sole voltage-gated sodium channel (Na_V_) in *Drosophila*, Para, is localized to the dendrites of CNs. Para is localized to the distal tip of the dendrites in all CNs, from embryos to adults, and is colocalized with the mechanosensitive TRP channels No mechanoreceptor potential C (NompC) and Inactive/Nanchung (Iav/Nan). Para localization also demarcates spike initiation zones (SIZs) in axons and the dendritic localization of Para is indicative of a likely dendritic SIZ in fly CNs. Para is not present in the dendrites of other peripheral sensory neurons. In both multipolar and bipolar neurons in the PNS, Para is present in a proximal region of the axon, comparable to the axonal initial segment (AIS) in vertebrates, 40–60 μm from the soma in multipolar neurons and 20–40 μm in bipolar neurons. Whole-cell reduction of *para* expression using RNAi in CNs of the adult Johnston’s organ (JO) severely affects sound-evoked potentials (SEPs). However, the duality of Para localization in the CN dendrites and axons identifies a need to develop resources to study compartment-specific roles of proteins that will enable us to better understand Para’s role in mechanosensitive transduction.

## Significance Statement

Several transient receptor potential (TRP) channels have been shown to localize to dendrites of *Drosophila* mechanosensitive chordotonal neurons (CNs). Here, we show that the fly voltage-gated sodium channel (Na_V_), Para, co-localizes with the TRP channels NompC and Iav and a possible dendritic spike initiation zone (SIZ) in CN dendrites. This dendritic localization is unique to CNs, is not seen in other peripheral neurons, and may account for some aspects of mechanotransduction. Para also localizes to a SIZ at an axonal initial segment (AIS)-like region, which is shared among many peripheral neurons.

## Introduction

Animals need to sense their environment to move, find food, and avoid predators. Peripheral nervous system (PNS) neurons are responsible for detecting environmental cues and relaying this information to the CNS. *Drosophila melanogaster* has provided important insights into sensory information processing in the PNS (Cosens and Manning, 1969; Göpfert et al., 2006; Akitake et al., 2015). The fly PNS contains multipolar and bipolar neurons (Bodmer and Jan, 1987; Orgogozo and Grueber, 2005). Multipolar neurons have one axonal neurite and many dendrites (Grueber et al., 2001; X. Wang et al., 2015). The expansive dendritic tree provides broad coverage of the animals’ periphery where transient receptor potential (TRP) channels open in response to pain, touch, and heat stimuli (Liu et al., 2003; Tracey et al., 2003; Zhong et al., 2010; Tsubouchi et al., 2012). Bipolar neurons have one axon and one dendrite separated by the soma (Orgogozo and Grueber, 2005). Bipolar neurons contain TRP channels sensitive to odors, chemicals, light, stretch, and sound (Stocker, 1994; Carlson et al., 1997; Vervoort et al., 1997; Ainsley et al., 2003; Schrader and Merritt, 2007; Suslak et al., 2015), and the singular dendrite enables the animal to precisely locate the direction of both attracting and deterring stimuli. Therefore, the TRP channel composition and orientation of neurons in the *Drosophila* PNS are optimized for sensing directional stimuli.

TRP channels alone do not account for all the electrophysiological properties of all sensory neuron dendrites. In *Drosophila*, a null allele for the mechanosensitive TRP channel *No mechanical potential C (NompC)*, which is localized to the most distal region of CN dendrites (Cheng et al., 2010; J. Lee et al., 2010), still has mechanosensitive properties in CNs (Eberl et al., 2000; Zhang et al., 2013). In addition to NompC, *Drosophila* CN dendrites also contain the TRP channels inactive (Iav) and nanchung (Nan) which form a functional heterodimer (Gong et al., 2004). Unlike NompC, Nan and Iav are required for the mechanosensitive response, however, in S2 cells the Nan-Iav complex alone is not mechanosensitive (B. Li et al., 2021). Hence, it is likely that not all the ion channels responsible for mechanotransduction in *Drosophila* are known.

In another invertebrate, the crayfish *Astacus astacus*, a model of the mechanosensitive response in stretch receptors using just TRP channels was unable to recapitulate *in vivo* recordings (Swerup and Rydqvist, 1996). However, when the models were altered to incorporate voltage gated sodium (Na_V_) channels, the recordings matched *in vivo* recordings indicating a possible mechanosensation role for Na_V_ channels (Suslak et al., 2011). Additionally, electrical spikes are present in mechanosensitive locust auditory neuron dendrites (Hill, 1983; Warren and Matheson, 2018) which are similar to the chordotonal neurons (CNs) in the Johnston’s organ (JO) in *Drosophila*. These spikes occur in the distal region of the dendrite and are sensitive to tetrodotoxin (TTX) suggesting a role for Na_V_s channels in mechanosensitive neurons (Hill, 1983; Warren and Matheson, 2018). Together this shows that mechanosensation in invertebrates does not just rely on mechanosensitive TRP channels and that Na_V_ channels may play a role in some peripheral neurons.

*Drosophila* has one Na_V_ channel gene encoded by *paralytic* (*para*; Suzuki et al., 1971). In the unipolar neurons of the CNS, *para* is expressed in active, mature neurons and is localized to the spike initiation zone (SIZ), where action potentials are generated (AP), at a distal axonal segment (DAS; Ravenscroft et al., 2020). Little is known about Para distribution in the multipolar and bipolar neurons of the PNS. Gene expression reporters in late embryos reveal *para* is expressed in some PNS neurons but it is unclear whether it is expressed in all or only some neurons (Hong and Ganetzky, 1994; Ravenscroft et al., 2020).

To determine the role of Na_V_ channels in mechanosensation in the *Drosophila* PNS we used a previously generated *Minos*-mediated integration cassette (MiMIC) protein trap inserted into the *para* locus to identify the distribution of Para (Venken et al., 2011; Lee et al., 2018; Ravenscroft et al., 2020). Using this allele, we identified the axonal SIZ of multidendritic and bipolar CNs in the third instar larval PNS in an axonal initial segment (AIS)-like region. Interestingly, we observe Para at the distal dendritic tip of all CN dendrites throughout development, indicating another role for Na_V_ channels in the peripheral mechanical response.

## Materials and Methods

### Fly lines and maintenance

Flies were raised on a standard molasses-based lab diet at 22°C in constant light conditions. All crosses were performed at 25°C in a 12-h light/dark incubator. Animals were not selected for sex at embryonic, larval, or pupal stages. Fly lines used are listed in Table 1. All fly lines used are either deposited in Bloomington *Drosophila* Stock Center, the Vienna *Drosophila* Resource Center, or are available on request. The characterization and validation of the gene-trap and protein-trap *para-alleles* were previously performed by Ravenscroft et al., 2020.

**Table 1:** Summary of fly lines used in this study

Fly line	Genotype	Stock source	Reference
*para-GFP*	*y^1^ w^67c23^ paraMI08578-GFSTF.0 Mi{PT-GFSTF.0}MI08578a Mi{PT-GFSTF.0}MI08578b*	BDSC #91528	Ravenscroft et al. (2020)
*para-RFP*	*y^1^ w^67c23^ paraMI08578-TRH.0 Mi{PT-TRH.0}MI08578a Mi{PT-TRH.0}MI08578b*	BDSC #92157	Ravenscroft et al. (2020)
*Para-T2A-GAL4*	*y1 w67c23 Mi{Trojan-GAL4.0}paraMI08578-TG4.0/FM7c*	BDSC #91527	Ravenscroft et al. (2020)
*221-GAL4*	w*; Pin^1^/CyO; P{?GawB}221w-	BDSC #26259	J. Huang et al. (2008)
*tilB-GAL4, nan-GAL4*	*w^1118^; tilB-Gal4 nan-Gal4*		Kim et al. (2003); Kavlie et al. (2010)
*nompB-GAL4*	*w^1118^; PBac{IT.GAL4}nompB2151-G4/CyO*	BDSC #65724	Gohl et al. (2011)
*ato-GAL4*	y^1^ w^*^; P{ato-GAL4.3.6}10	BDSC #9494	Hassan et al. (2000)
*UAS-RedStinger*	w*; P{w[+mC]=UAS-RedStinger}4, P{w[+mC]=UAS-FLP.D}JD1, P{w[+mC]=Ubi-p63E(FRT.STOP)Stinger}9F6/CyO	BDSC #28280	Evans et al. (2009)
*UAS-mCD8::RFP*	w*; P{y[+t7.7] w[+mC]=10×UAS-IVS-mCD8::RFP}attP40	BDSC #32219	Pfeiffer et al. (2010)
*UAS-mCherry*	y^1^ w*; wg[Sp-1]/CyO, P{Wee-P.ph0}Bacc[Wee-P20]; P{y[+t7.7] w[+mC]=20×UAS-6×mCherry-HA}attP2	BDSC #52268	Shearin et al. (2014)
*UAS-para-RNAi-GD3392*	*w^1118^; P{GD3392}v6131*	VDRC #6131	Dietzl et al. (2007)
*UAS-para-RNAi-GD3392*	*w^1118^;; P{GD3392}v6132*	VDRC #6131	Dietzl et al. (2007)
*UAS-para-RNAi-KK108534*	*P{KK108534}VIE-260B*	VDRC #104775	Dietzl et al. (2007)
*UAS-Dicer2*	*w^1118^; P{w[+mC]=UAS-Dcr-2.D}10*	BDSC # 24651	Dietzl et al. (2007)
Canton S	*Canton-S*	BDSC # 64349	

### Immunostaining

#### Embryos

Immunostaining of *Drosophila* embryos was done as described previously (Rothwell and Sullivan, 2007). Flies were crossed in a chamber containing a grape juice plate (Welch) at 22°C in constant light. Flies lay eggs predominantly around dusk; therefore, to collect embryos at stage 16 we waited for 20–24 h for collection. Embryos were collected with a paintbrush and water into a cell collection chamber (VWR #732–2758). These baskets were placed in a 50% bleach solution for dechorionation and agitated with a pipette. Dechorionation was observed under a microscope and once >75% of embryos lost the dorsal appendages the embryos were washed with an embryo wash solution (0.7% NaCl, 0.05% Triton X-100 in water). The basket was then dried by placing the chamber on a Kim wipe. For fixation, the mesh of the basket was removed with a razor blade and placed in a glass scintillation vial. The mesh was washed with 1 ml of heptane saturated with 37% formaldehyde (equal volumes of heptane and 37% formaldehyde were placed in a scintillation vial and vigorously mixed several times, the solution was allowed to settle into two phases with saturated heptane in the upper phase) which removed the embryos from the mesh. The mesh was then removed and 1 ml of 3.7% formaldehyde in PEM buffer (0.1 m PIPES, 1 mm MgCl_2_, and 1 mm EGTA, pH 6.9, in water) was added, the vial was vigorously mixed for 15 s, and then left at room temperature for 20 min. The bottom formaldehyde layer was then removed and replaced with methanol (100%), this mixture was vigorously mixed for 15 s and left to stand for 1 min. After the embryos sank to the bottom of the vial and the upper heptane layer was removed. The vial was then filled ∼2/3 full of 100% methanol and left to sit at 4°C overnight. Embryos were then transported to a 1.5 ml Eppendorf tube. As much methanol as possible was removed and replaced with 500 μl of PBTA (1× PBS, 0.05% Triton X-100, 0.02% sodium azide). They were then placed on a rotator at room temperature for 15 min to rehydrate. Primary antibodies were then incubated in the vials in PBTA and left at 4°C overnight on a rotator. Antibodies were then recovered, and embryos were rinsed three times with PBTA and left for 1 h in PBTA on a rotator. Secondary antibodies were then added in PBTA and incubated on a rotator at room temperature for 2 h (wrapped in foil to avoid light exposure). Antibodies were then recovered, and embryos were rinsed three times with PBTA and left for 1 h in PBTA on a rotator. Embryos were rinsed four times with PBS-Azide (1× PBS, 0.02% sodium azide) to remove the detergent. Embryos were mounted in rapiclear 1.47 (SUNjin labs) for imaging. Embryos were placed on a glass slide with a coverslip with no spacer. Antibodies used were rabbit-GFP 1:200 (Invitrogen #A-11122), rat-Elav 1:500 (DSHB #7E8A10), mouse-FLAG 1:200 (F3165 Sigma), rabbit-NompA 1:200 (Chung et al., 2001). Secondary antibodies used were goat-anti-HRP-Cy3 1:500 (Jackson ImmunoResearch, #123-165-021) and corresponding donkey secondary antibodies 1:500 (Jackson ImmunoResearch).

#### Third instar larvae

Wandering third instar larvae were placed in cold 1× Schneiders medium (SM). Larvae were pinned on a Sylgard plate with Minuten pins in the anterior and posterior of the animal, dorsal side down. An incision was made with fine dissection scissors from the posterior to the anterior of the animal. Internal organs and fat were removed from the animal. Pins were placed to fillet the larvae. Three to four animals at a time were filleted on each plate. The SM was then replaced with 3.7% paraformaldehyde (PFA) in SM and placed on a gentle rocker for 20 min at room temperature. After 20 min larvae were rinsed three times with SM, pins were removed and larvae were placed in a micro-Eppendorf tube in 0.1% PBS-TX (1× PBS, 0.1% Triton 100-X), tubes were washed three times for 10 min on a rotator at room temperature. Primary antibodies were then added and incubated with 0.1% PBS-TX overnight on a rotator at 4°C. Antibodies were then recovered, and animals were rinsed three times with 0.1% PBS-TX and then washed three times for 10 min in 0.1% PBS-TX. Secondary antibodies were then added and incubated (wrapped in foil) for 2 h at room temperature on a rotator. Antibodies were then recovered, and animals were rinsed three times with 0.1% PBS-TX and then washed three times for 10 min in 0.1% PBS-TX. Larvae were then mounted on a glass slide in rapiclear 1.47 (SUNjin labs) under a coverslip with no spacer. Antibodies used were rabbit-GFP 1:200 (Invitrogen #A-11122), mouse-GFP 1:200 (Sigma G6539), rabbit-NompA 1:500 (Chung et al., 2001), mouse-Eys 1:50 (DSHB #21A6 (Fujita et al., 1982)], and rabbit-NompC 1:300 (Cheng et al., 2010). Secondary antibodies used were goat-anti-HRP-Cy3 1:500 (Jackson ImmunoResearch, #123-165-021) and corresponding donkey secondary antibodies 1:500 (Jackson ImmunoResearch). The specificity of the anti-GFP antibody for Para-GFP is shown in Extended Data Figure 4-1.

Intensity profiles for Para distribution were calculated using a previously published approach (Jegla et al., 2016). Stacked confocal images of ddaE neurons were processed in ImageJ, a line was measured from the soma along the axon as far as a single axon track could be followed. The intensity of UAS-mCherry and GFP staining was recorded using the measurement feature. Relative intensity was measured by dividing the measured value by the average of the lowest 20% of measurements. This value was then divided by the top 5% of values (after dividing by the lowest 20%) to give a relative intensity. This was performed on *n* = 15 neurons from *n* = 5 animals. Measurements from all animals were then combined, smoothed out to an average of 50 values, and plotted on a graph. GFP to mCherry ratio was measured by comparing each relative GFP measurement to the corresponding mCherry measurement. For CN neurons the measurements were started at the distal tip of the dendrite through the soma into the axon. These data were represented with the soma at 0 using the max intensity point of mCD8::mCherry as the soma. Because of variation in dendrite length between animals, a representative trace is shown.

##### Johnston’s organ

Johnston’s organ dissections and imaging were performed as described previously (T. Li et al., 2016). Pupae 24–48 h after puparium formation were placed on double-sided tape on a glass slide. Using forceps, the outer shell was removed, and the pupae were removed and placed in a Sylgard dish in SM. Using micro scissors, the head was removed. A pipette was used to provide suction and remove the fat and the brain from the head, leaving the antenna attached to the outer membrane. This membrane was fixed in 3.7% PFA in PBS for 20 min, then rinsed three times and washed three times for 10 min in 0.1% PBS-TX. Samples were incubated in conjugated antibodies in 0.1% PBS-TX and incubated overnight at 4°C. Antibodies were recovered and samples were rinsed three times and washed three times for 10 min in 0.1% PBS-TX. Samples were mounted in Vectashield on a glass slide with 2 pieces of double-sided tape acting as a spacer to protect the antennae. Antibodies used were rabbit-GFP-488 1:200 (Invitrogen), and goat-phalloidin-Cy3 1:500 (Jackson ImmunoResearch).

### Johnston’s organ electrophysiology

Sound-evoked potential (SEP) recordings were performed with an electrolytically sharpened tungsten recording electrode inserted into the joint between antennal segments one and two, and a reference electrode inserted into the head cuticle near the posterior orbital bristle, in response to near-field playback of computer-generated pulse song (described by Eberl and Kernan, 2011). The signals were subtracted and amplified with a differential amplifier (DAM50, World Precision Instruments) and digitized at 10 kHz (USB-6001, National Instruments). Average response values were measured as the max-min values in an averaged trace from 10 consecutive presentations of the described protocol.

### Experimental design

All confocal images of embryos were correctly aged using the structure of the CNS labeled by horseradish peroxidase (HRP). JO images were taken from pupae 48–72 h after puparium formation. Adult flies aged 2–7 d were used for JO electrophysiology. Images from more than five animals for each condition were obtained.

### Statistical analysis

A one-way analysis of variance (ANOVA) with Brown–Forsythe correction for unequal standard deviations was used for the comparison of SEP in JO electrophysiology data. Graphs indicate, within each bar, the number of antennae tested for that genotype. Statistical significance depicted on graphs indicate Tukey’s *post hoc* multiple comparisons test.

## Results

### *para* is expressed in multidendritic neurons and CNs in *Drosophila* embryos

**Figure 1.**
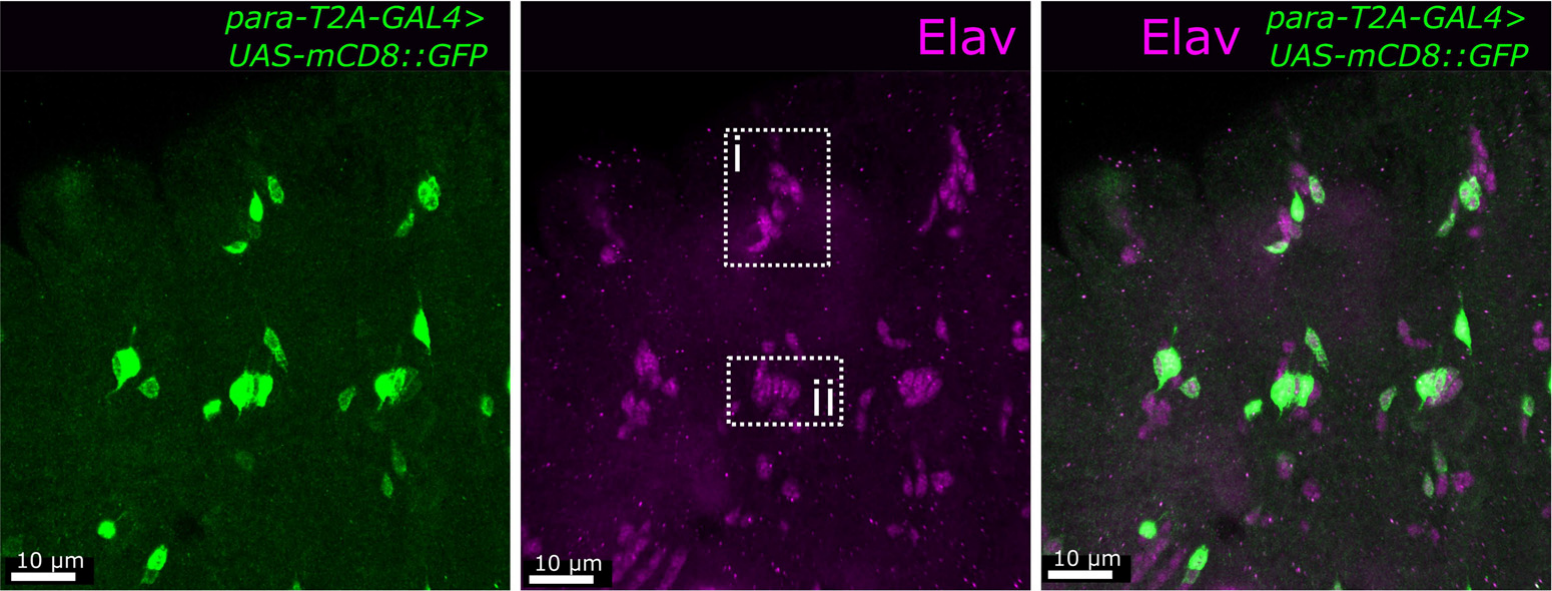
*para* is expressed in embryonic chordotonal neurons. The *para-T2A-GAL4* allele combined with a UAS-mCD8::GFP enables the visualization of *para*-expressing cells. In stage 16 embryos, where *para* is first expressed, *para* is expressed in a restricted number of Elav-positive PNS neurons including all chordotonal neurons (box ii). *para* is expressed in some multidendritic neurons (box i).

*para* expression is first noted at stage 16 of embryonic development where it is expressed in some CNS neurons (Hong and Ganetzky, 1994; Ravenscroft et al., 2020). The *para-T2A-GAL4* allele, when paired with a fluorescent reporter (*UAS-mcD8:GFP*), labels the cells that express *para* (Ravenscroft et al., 2020). We used *para-T2A-GAL4* to determine the expression pattern of *para* in the PNS in stage 16 embryos. The PNS of embryos contains developing multipolar and bipolar neurons responsible for mechanosensation, proprioception, temperature, and touch (Orgogozo and Grueber, 2005). Multipolar neuron cell bodies have multiple dendritic processes with one axon, whereas bipolar neuron cell bodies have only one dendrite and one axon. These neurons can be easily identified by their location on the embryo (Singhania and Grueber, 2014): the dorsal cluster of neurons (Fig. 1*A*, box ii) are all multipolar, a medial cluster of 5 neurons lined up parallel to each other are bipolar lateral chordotonal neurons (lch5; Fig. 1*A*, box i). Like in the CNS (Ravenscroft et al., 2020), *para* is not expressed in all PNS neurons (Fig. 1*A*) as *para* is expressed in a restricted number of multipolar neurons (Fig. 1*A*, box i). Unlike the multipolar neurons, *para* is expressed in all the bipolar CNs (Fig. 1*A*, box ii). Note that muscle cells in the embryo are also not labeled with *para, in* contrast to vertebrates where Na_V_ channels are needed for muscle contraction (George et al., 1991).

### Para is localized to embryonic CN dendrites and soma

To determine Para localization, a MiMIC converted *para*-allele containing multiple epitopes for antibody labeling [Para-GFP-FlASH-Strep-TEV-FLAG (further referred to as Para-GFP)] was used (Venken et al., 2011; Ravenscroft et al., 2020). The Para-GFP allele has been previously validated and characterized to be representative of endogenous Para localization (Ravenscroft et al., 2020). We tested where Para is localized in the neurons relative to Elav, which marks the soma of neurons, and HRP, which labels all neuron membranes.

**Figure 2.**
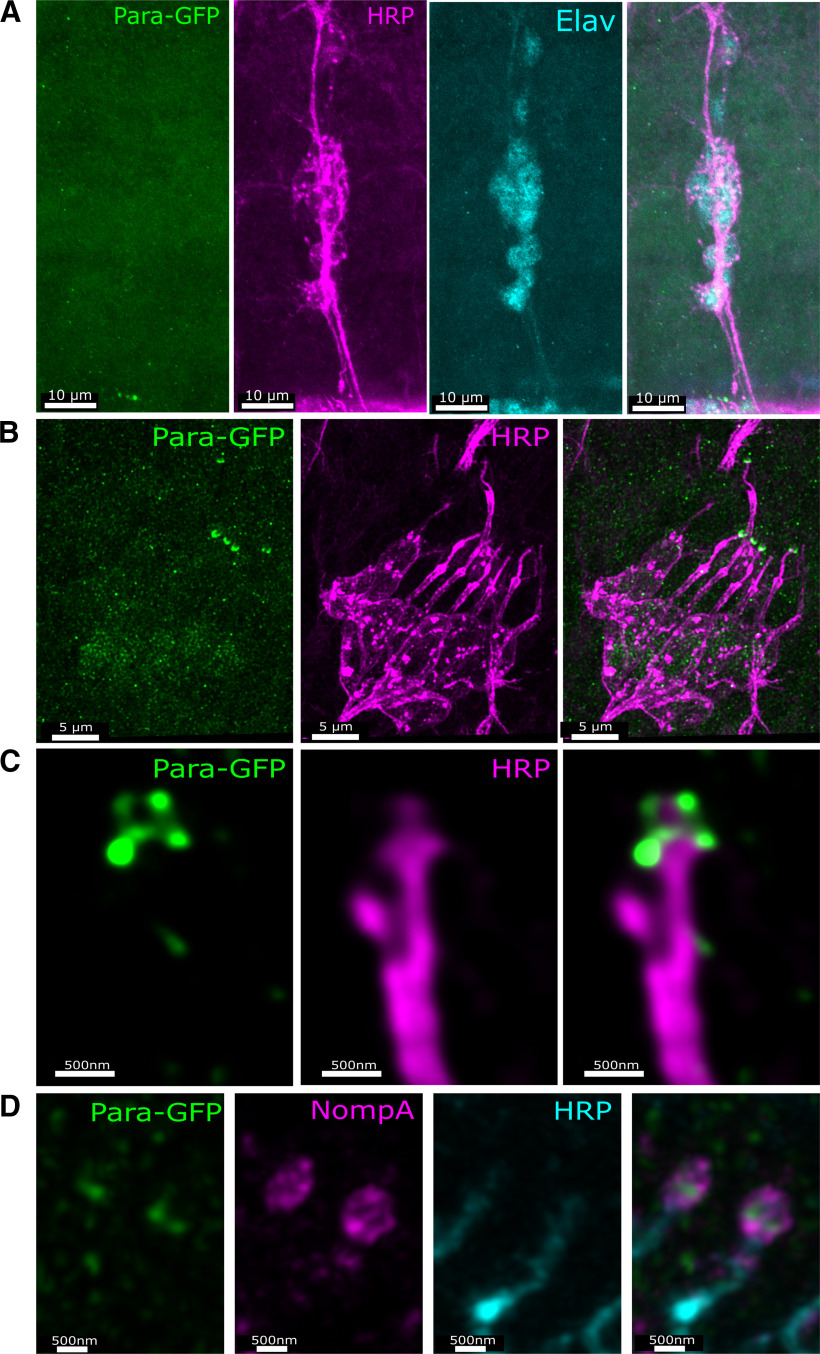
Para is enriched in the distal dendrites of embryonic chordotonal neurons. Despite *para* expression in multidendritic neurons (Fig. 1), the Para protein cannot be seen in axons, the soma, or dendrites indicating expression is very low (***A***). In the chordotonal neurons, Para is seen predominantly in the dendrites with less Para in the soma and no observed Para in axons. Neuronal membranes are labeled with an antibody against horseradish peroxidase (HRP; ***B***). Para is localized to the very distal tip of the dendrite (***C***), where it is surrounded by the dendritic cap protein NompA (***D***).

In stage 16 embryonic multidendritic neurons, Para-GFP is not observed in the axons, dendrites, or soma (Fig. 2*A*). At this stage the multidendritic neurons are still developing (Bodmer and Jan, 1987; Hartenstein, 1988); however, unlike the developing motor neurons of the CNS where Para is localized to the soma (Ravenscroft et al., 2020), Para is not detected in multidendritic neurons during development. This indicates Para’s functional role in these neurons occurs at later stages of development. In contrast, CNs become fully differentiated in stage 16 of embryonic development (Jarman et al., 1993). *para* is strongly expressed in all embryonic CNs at stage 16. Para is predominantly localized to the distal tip of the dendrites with a lower level of Para seen in the soma (Fig. 2*B*). Super-resolution stimulated emission depletion (STED) microscopy revealed that Para is present at the very distal tip of the dendrite with no membrane labeling via HRP present beyond where Para is localized (Fig. 2*C*). No Para is detected in the axon at this stage. The distal tip of the CN dendrite is connected to a dendritic cap via an extracellular matrix (ECM) that includes the glycoprotein NompA. NompA is specifically expressed in type I sense organs by support cells (scolopale cells) that ensheath the sensory process (Chung et al., 2001). In the distal CN dendrite of stage 16 embryos, Para is surrounded by NompA (Fig. 2*D*).

### Para is expressed in all PNS neurons in third instar larvae and enriched at an AIS-like region in axons of multipolar neurons

**Figure 3.**
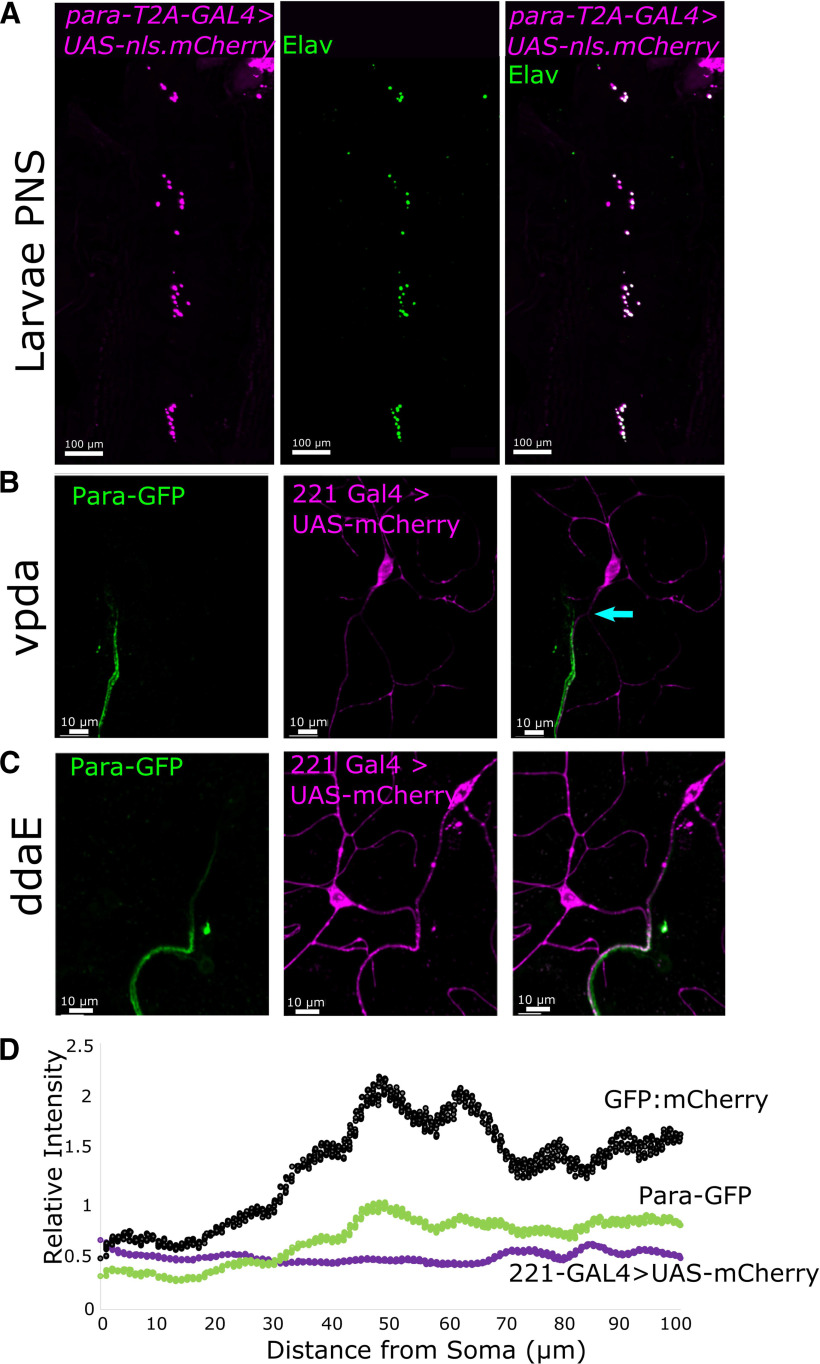
Para is localized to an AIS-like region in multipolar neurons of the third instar larvae PNS. *para* is essential for larval development and hence the expression and localization of Para in larval stages likely indicate where it functions. In the third instar larvae, PNS *para* is expressed in all neurons (Elav-positive cells; ***A***). In the third instar larvae multidendritic PNS neurons, Para is localized to the proximal axon in both vpda (***B***) and ddaE (***C***) neurons. In vpda neurons, a dendrite can be seen in the proximal axon (arrow), Para is localized distal to this dendrite. In ddaE neurons, the relative intensity of Para localization is highest ∼40–60 μm from the soma indicating the presence of an axonal initial segment-like region in the third instar larva PNS (***D***). Beyond the AIS-like region, Para is still present at lower levels, likely to maintain the propagation of depolarizations to the synapse.

We assessed the expression pattern in the third instar larval stage using *para-T2A-GAL4*. *para-T2A-GAL4* expression of *UAS-nls.mCherry* (Evans et al., 2009) shows that *para* is expressed in all Elav-positive neurons in the PNS (Fig. 3*A*). This contrasts with the expression of *para* in the third instar larvae CNS where it is only present in ∼25% of neurons (Ravenscroft et al., 2020).

In the third instar larva and adult CNS Para is localized to a DAS while in stage 16 embryos Para is localized to the soma of neurons (Ravenscroft et al., 2020). Most CNS neurons in *Drosophila* are unipolar, while PNS neurons are either multipolar (multidendritic neurons) or bipolar (CNs). To determine Para localization in fully developed PNS neurons we used Para-GFP in combination with 221-GAL4 (Huang et al., 2008), which drives GAL4 expression in some multipolar PNS neurons including ventral dendritic arborization (vpda) and dorsal dendritic arborization (ddaE) neurons (Grueber et al., 2003), and UAS-mCherry (Shearin et al., 2014) in wandering third instar larva. In both the multidendritic vpda (Fig. 3*B*) and ddaE neurons (Fig. 3*C*), like CNS neurons, Para is localized to the axon but not the soma or dendrites. However, unlike in the CNS, Para is enriched in a segment that is only 40–60 μm from the soma (Fig. 3*D*). This region overlaps with a previously reported AIS-like region marked by the localization of overexpressed Ankyrin 2 (Ank2) isoforms but is distal to the localization of overexpressed voltage-gated potassium (K_V_) channels Eag-like K^+^ channel (Elk) and Shaker cognate I (ShaI) (Jegla et al., 2016). Beyond the AIS-like region, Para is still present at lower levels. We speculate that continued Para distribution is needed to maintain AP propagation beyond the initiation site. Additionally, in the vpda neuron, we observe dendrites that enter the axons beyond the cell body (Fig. 3*B*). This branching has been previously reported to occur in some vpda neurons (Schrader and Merritt, 2000). Similar to Para localization at a DAS in CNS neurons, Para is localized distal to the axonal dendrite in the vpda neurons.

### In the larva, PNS Para is localized to axons and dendrites of CNs

**Figure 4.**
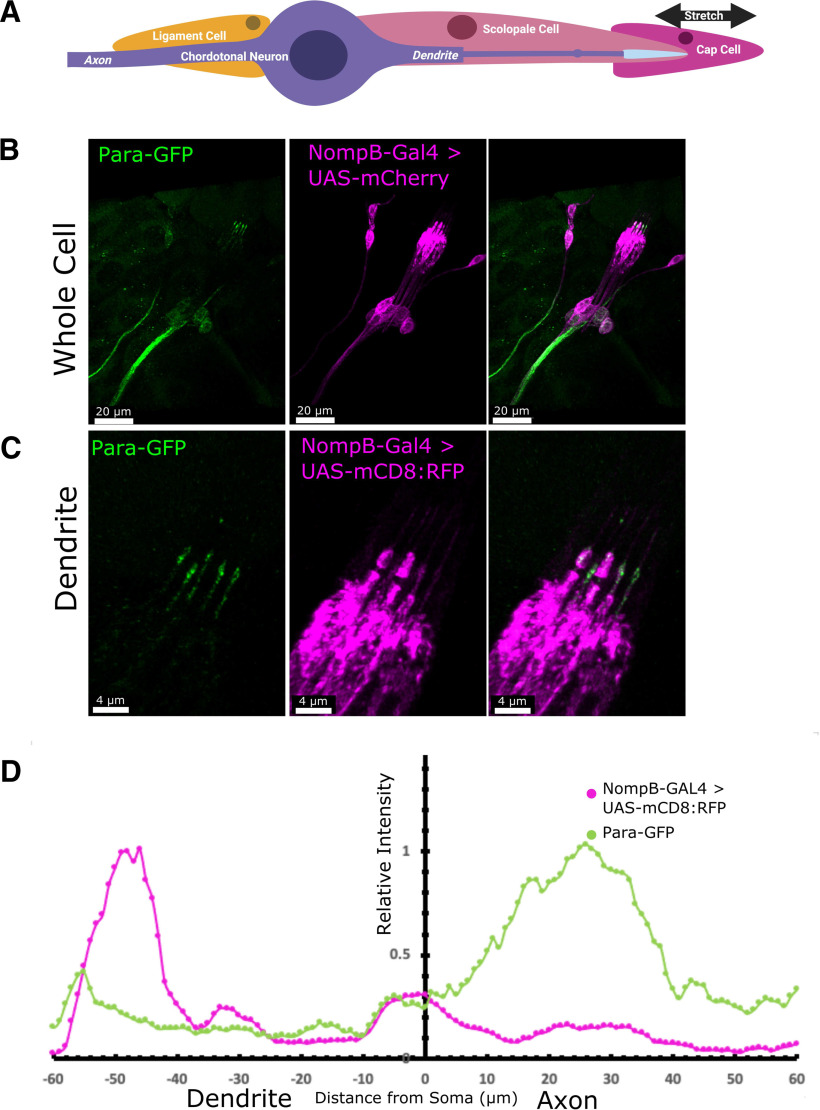
Para is localized to both axons and dendrites in third instar larval chordotonal neurons. The chordotonal neuron is part of a 4-cell chordotonal organ (***A***). The neuron is anchored at the soma by a ligament cell and at the dendrite by the cap cell. The dendrite is insulated by a scolopale cell that provides structural support and protects the ionic balance of the dendritic space. NompB-GAL4 is expressed in the chordotonal neurons of the third instar larval PNS (Gohl et al., 2011) and with UAS-mCD8::RFP (Pfeiffer et al., 2010) these neurons can be visualized (***B***). Para is enriched in both the axon and dendrite of the lch5 neuron (***B***). In the axon, Para is localized ∼20–30 μm proximal to the soma, closer than what is observed in multidendritic neurons (***D***). In the dendrites (***C***), Para is localized to the distal dendrite ∼50–60 μm from the soma (***D***). The distance on the axis in ***D*** indicates the distance from the soma into the dendrite (0 to −80 μm) and the axon (0 to +80 μm). The specificity of the anti-GFP for Para-GFP is shown in Extended Data Figure 4-1.

CNs are part of a four-cell chordotonal organ containing a neuron, a ligament cell that anchors the neurons, a cap cell that is attached to the CN dendrite via the dendritic cap, and a scolopale cell that protects and maintains the environment around the CN dendrite (Fig. 4*A*; Hartenstein, 1988). CN neurons and dendrites, like multidendritic neurons, continue to stretch and grow as the larvae grow (Singhania and Grueber, 2014). In wandering third instar larvae, the CNs reach their maximum length. In the lch5 CNs in the larval abdomen, Para is enriched in axons (Fig. 4*B*). As in multidendritic neurons, Para in CNs is localized in the proximal part of the axon but unlike multidendritic neurons, Para is enriched close to the soma (20–30 μm; Fig. 4*D*). Additionally, the drop-off in the intensity of Para localization beyond the AIS-like region is greater in the CNs than in the multidendritic neurons. Hence, the AIS-like region is present in bipolar PNS cells as well as multipolar cells.

10.1523/ENEURO.0105-23.2023.f4-1 Extended Data Figure 4-1 The A-11122 (Invitrogen) anti-GFP anti-body is specific for Para-GFP and does not label the dendrite, soma, or axon of lch5 neurons in Canton S animals. Download Figure 4-1, TIFF file.

**Figure 5.**
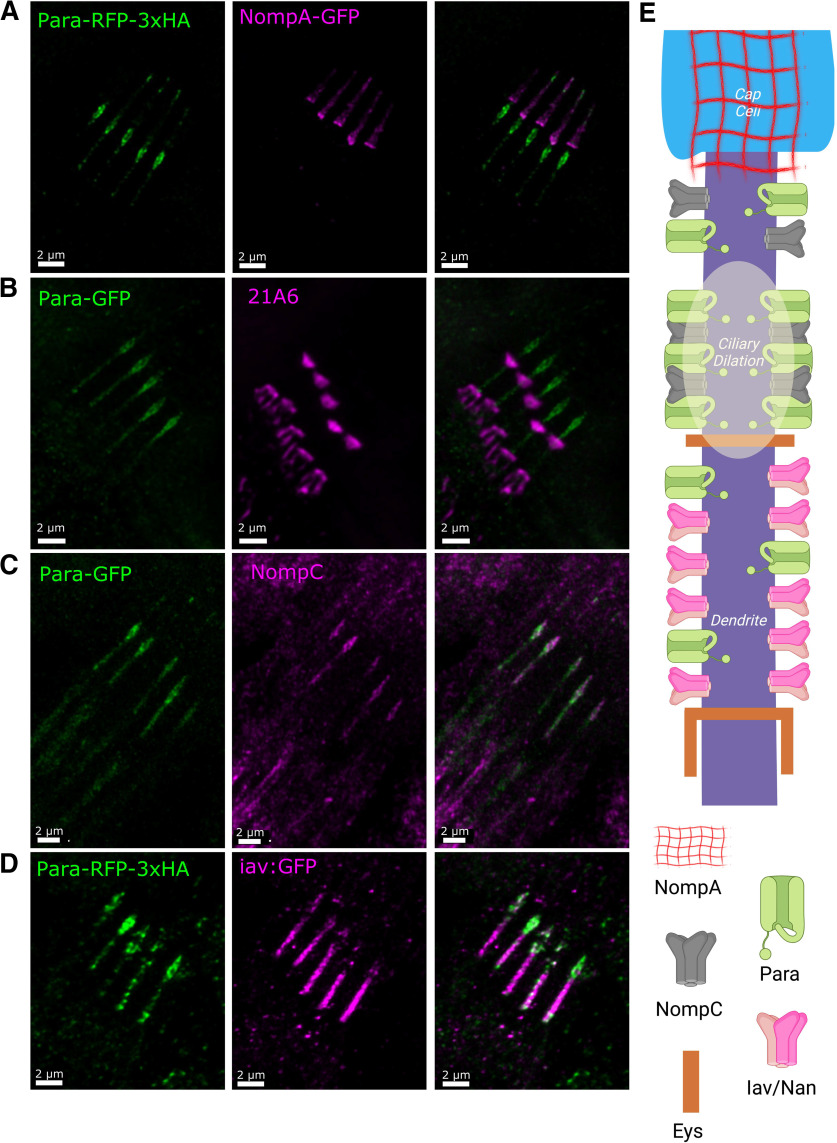
Para colocalizes with NompC in the ciliary dilation and distal dendrite of third instar larva chordotonal neurons. Several proteins have been localized to dendrites of chordotonal neurons (***E***). NompA anchors the chordotonal dendrite to the dendritic cap. Para is localized to two segments of the dendrite, the most distal segment overlaps with NompA binding to the distal tip (***A***). The more proximal region of Para is on the proximal side of the dendrite where NompA is localized, distal to the scaffold protein Eyes-shut (labeled with Mab 21A6; ***B***). This location corresponds to ciliary dilation. The mechanosensitive transient receptor potential (TRP) channels NompC, Iav, and Nan are all localized to the dendrites of chordotonal neurons with NompC in the distal region and Iav and Nan proximal to the ciliary dilation. Para colocalizes with both NompC and Iav (***C***, ***D***); however, Para is more abundant where NompC is localized. The localization of each protein is summarized in ***E***.

In the dendrites of the third instar larval lch5 CNs, like in embryonic CNs, Para is localized to the distal tip (Fig. 4*C*). Para is less abundant at the dendrite than it is at the axon (Fig. 4*D*). When compared with the localization of the cap protein NompA (Chung et al., 2001), Para is enriched both distal and proximal to the cap (Fig. 5*A*). The more proximal localization of Para overlaps with the ciliary dilation which can be seen by the expansion of the dendrite distal to the extracellular scaffolding protein eyes-shut (Eys; Fig. 5*B*; Blochlinger et al., 1991; Husain et al., 2006). Low levels of Para can also be seen proximal to the ciliary dilation. The dendrites of the CNs contain the mechanosensitive ion channels NompC, and the two interdependent TRP channels Iav and Nan (Nan; Zhang et al., 2013). NompC is localized at the ciliary dilation and at the tip of the dendrite, while Iav and Nan are localized proximal to the ciliary dilation (Fig. 5*E*; Gong et al., 2004; J. Lee et al., 2010; Liang et al., 2011). Para predominantly colocalizes with NompC at the tip of the dendrite (Fig. 5*C*). Para also partially colocalizes with the more proximal Iav (Fig. 5*D*); however, the majority of Para in dendrites is at the ciliary dilation together with NompC.

### Para is required for sound response in the Johnston’s organ CNs

**Figure 6.**
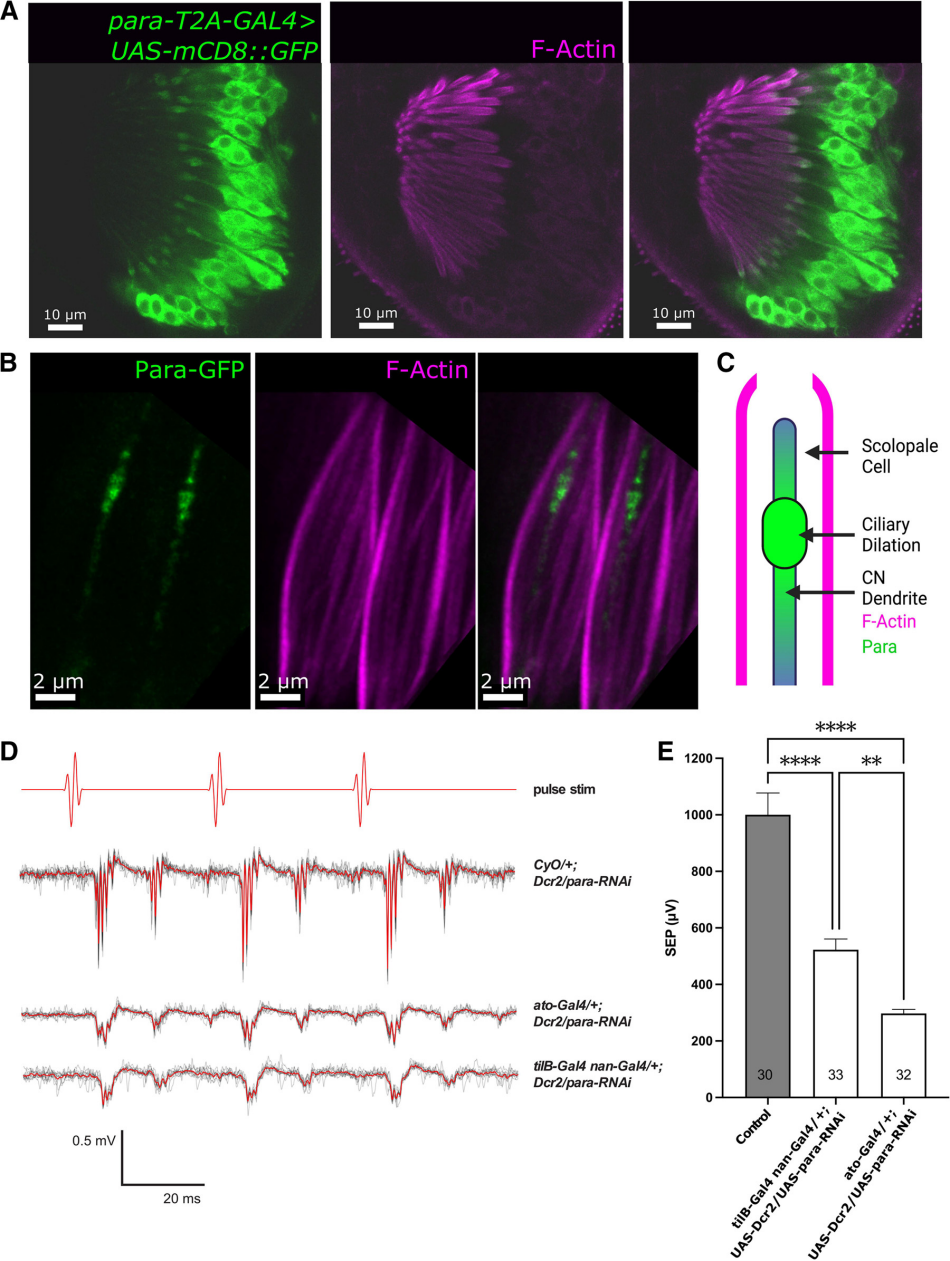
Para is required for sound response in Johnston’s organ (JO). The JO is responsible for detecting auditory stimuli in the adult fly. In the developing pupae, ∼54 h after puparium formation *para* is expressed in the chordotonal neurons (CN) of the second antennal segment and not in the support scolopale cells labeled by F-Actin (***A***). In the chordotonal neurons, Para is localized to the ciliary dilation and distal dendrite of the chordotonal neurons in the JO (***B***). Diagram depicting the spatial arrangement of Para relative to the sensory cilium and scolopale rods (***C***). The activity of adult chordotonal neurons can be measured using sound-evoked potentials (SEPs). Acoustic near-field presentation of computer-generated pulse song stimulus (red pulse stim line) evokes strong SEPs in control animals with no Gal-4 driver. Responses to 10 individual stimulus presentations (depicted as light gray lines) are averaged (depicted as red lines; ***D***). RNAi knock-down of *para* using the *ato-Gal4* driver, which drives expression in the chordotonal sense organ precursor and the resulting chordotonal lineage, or with the *tilB*-Gal4 *nan*-Gal4 driver, which drives expression only in the chordotonal neurons, with Dicer-2 (Dcr2) results in a strong reduction in the SEP amplitude (***E***). The SEP of multiple knock-down lines is shown in Extended Data Figure 6-1. Bars and error bars indicate mean ± SEM, and *N*s shown within each bar indicate the number of antennae tested. Results of one-way ANOVA with Brown–Forsythe correction and Tukey’s *post hoc* multiple comparisons test are shown (**p < 0.01, ****p < 0.0001).

Adult flies have a greater sensory repertoire than larvae. Adult flies have more sensitive responses to sound stimuli and have a greater abundance of proprioceptive and mechanosensitive neurons to maintain complex functions such as flight stabilization and courtship (Currier and Nagel, 2020; Montell, 2021). CNs are essential for hearing in adult *Drosophila* (Eberl et al., 2000). The second antennal segment of the adult fly contains the JO, which consists of 225 scolopidia (Caldwell and Eberl, 2002; Kamikouchi et al., 2006). Each scolopidium contains 2 or three bipolar CNs anchored in antennal segment 2 (Todi et al., 2004). The dendrites are attached to a tubular ECM dendritic cap anchored to the rotating stalk of antennal segment 3 (Todi et al., 2004). The neuron is surrounded by a scolopale cell, a glial-like cell that protects the dendrite and maintains the ionic balance of the extramembrane space of the dendrite (Caldwell and Eberl, 2002; Roy et al., 2013). When sound waves reach the antenna, they cause the stalk to rotate and pull on the dendrites of CNs, opening the mechanosensitive TRP channels NompC, Iav, and Nan and initiating a graded potential (Göpfert et al., 2006). The JO develops during pupal stages. Pupae 24–48 h after puparium formation were dissected to observe Para localization and expression. Using *para-T2A-GAL4* driving expression of *UAS-mCD8::GFP*, we do not observe any scolopodia, as labeled by F-Actin, that are not connected to a *para* expressing cell, therefore, like the CN in larvae and embryos, *para* is expressed in all the CNs of the JO (Fig. 6*A*). This is consistent with the FlyCellAtlas which shows *para* expression in all JO neurons (H. Li et al., 2022). Each scolopodium contains two or three JO neurons that are encased by the F-Actin spindles (Todi et al., 2004). Para is also enriched at the ciliary dilation of the dendrite in JO neurons; however, in each scolopidium, only one Para containing dendrite is observed (Fig. 6*B*,*C*; Movie 1). Each scolopodium contains a stereotyped combination of neurons for detecting sound, wind, and gravity respectively (Ishikawa et al., 2020). The restriction of Para localization to just one neuron’s dendrite indicates a different physiological role of Na_V_ channels in different JO neuron types.

10.1523/ENEURO.0105-23.2023.f6-1 Extended Data Figure 6-1 Multiple *para* RNAi lines reduce SEP when expressed in Johnston’s Organ chordotonal neurons. Electrophysiological tests as in Figure 6 show significantly reduced SEPs when driving three different RNAi lines (6131, 6132, 104775) using two different Gal4 drivers (a chromosome carrying both tilB-Gal4 and nan-Gal4, and ato-Gal4), all in the presence of UAS-Dicer2 (indicated by +). Symbols and statistics as in Figure 5 (ns, not significant; *p > 0.5, ****p < 0.0001). Download Figure 6-1, TIFF file.

Movie 1. Para is only present in one Johnston’s Organ neuron per scolopodium. Magenta, F-Actin; green, Para-GFP 10.1523/ENEURO.0105-23.2023.video.1

To determine whether *para* is essential for CN mechanosensation in the adult JO, we used three RNAi lines (Dietzl et al., 2007) against *para* driven by two separate CN drivers: *atonal-Gal4* (*ato-GAL4*) (Hassan et al., 2000), which drives GAL4 expression in CN precursor cells and the chordotonal lineage, and *tilB-Gal4 nan-GAL4*, which drives GAL4 expression only in CNs (Kim et al., 2003; Kavlie et al., 2010; Roy et al., 2013). All RNAi lines target an exon incorporated into all 60 *para* isoforms ensuring we are not looking at isoform-specific effects (Larkin et al., 2021).

When *para* expression is reduced using any of the three RNAi lines against *para* with either GAL4 driver, the sound-evoked potentials (SEPs) produced in adult female flies in response to computer-generated male courtship pulse song is greatly reduced compared with control animals with no GAL4 driver (Fig. 6*D*,*E*; Extended Data Fig. 6-1). Additionally, when *para* expression is reduced, the SEPs in response to pulse stimulation are strongly reduced (Fig. 6*D*,*E*). Interestingly a small depolarization can still be detected in the neurons, possibly from the mechanosensitive channels that are remaining (NompC, Iav, Nan). However, when the *para* expression is reduced this signal is severely diminished implicating a role for Para in the excitability of the CN dendrite. Because of the dual nature of Para localization, the reduction in SEPs is likely the result of the loss of *para* in both axons and dendrites. However, we have not been able to isolate the dendrite or axonal-specific functions of Para as we have not been able to remove Para only in dendrites. Attempts were made to inhibit Para using the sodium channel blocker tetrodotoxin, however, we were not able to access the CNs likely because of the glial sheath (Nelson and Laughon, 1993).

## Discussion

In *Drosophila*, the composition of ion channels that contribute to the graded potentials in PNS cell dendrites is unclear. Mapping the distribution of Na_V_ channels in the unipolar neurons of the fly CNS uncovered the SIZ at a DAS (Ravenscroft et al., 2020). Using the same endogenously tagged Para allele, we located the likely SIZ in the multipolar and bipolar neurons of the *Drosophila* PNS. In contrast to the DAS in the unipolar neurons of the fly CNS, the SIZ is at an AIS-like region proximal to the soma in PNS, comparable to the location of the AIS in vertebrate neurons (C.Y.M. Huang and Rasband, 2018). Despite the more proximal location, the SIZ still determines the boundary between the somatodendritic and axonal compartments of the cell. Surprisingly, in addition to the axonal SIZ, a dendritic SIZ, demarcated by the presence of Na_V_ channels, is present in bipolar CN neurons. The dendritic SIZ is located at the distal tip of the dendrite and overlaps with the localization of the mechanosensitive TRP channels NompC and Iav. We believe this is a dendritic SIZ in agreement with a computational model of the crayfish stretch receptors for which Na_V_ activation is needed to accurately represent *in vivo* recordings (Suslak et al., 2011).

The localization of Para to the CN dendritic SIZ likely explains the TTX-sensitive dendritic spikes previously reported in insect mechanosensitive neurons (Hill, 1983; Oldfield and Hill, 1986; Lehnert et al., 2013; Warren and Matheson, 2018). Two TTX-sensitive spikes occur in the dendrites of locust auditory neurons dendrites. These spikes are recorded in the apical (distal) and basal (proximal) dendrite (Hill, 1983). The basal spikes respond to axonal depolarization and are likely backpropagating APs originating from Para channels opening at the SIZ in the axon. The apical spikes were of unknown origin but are likely to be spikes initiated by Para at the dendritic SIZ. While the dendritic SIZ identified in this study is in the fly and not the locust, Na_V_ localization is comparable between insect species as indicated by grasshopper Para and *Drosophila* Para having similar localization patterns (H. Wang et al., 2020).

All three identified TRP channels in the CN dendrites, NompC, Iav, and Nan, contribute to the mechanotransduction response. Direct patch clamp recordings of lch5 neurons identify a complete loss of mechanotransduction in the absence of Iav and Nan, while loss of NompC did not decrease the mechanotransduction response indicating that Iav and Nan are the essential channels (B. Li et al., 2021). Patch clamp experiments did uncover that without NompC the adaptation time in CNs is a lot shorter (B. Li et al., 2021), therefore the interplay between TRP channels in the dendrites is key for their proper function. The presence of Para in the same dendritic space as both NompC and Iav would enable Para to facilitate this interaction. It is worth noting, however, that these patch-clamp experiments were performed in conditions where Na_V_ was inhibited, therefore the electrophysiological interplay between Para and the TRP channels remains to be established.

In lch5 and JO CNs, Para is enriched at the ciliary dilation. The ciliary dilation is visible via a bulge in the membrane and is necessary for the separation of the dendrite into distinct regions (E. Lee et al., 2008). Beyond the ciliary dilation’s role in cellular organization, a role in signal transduction has been proposed but how this structure facilitates signal transduction is not clear (Moran et al., 1977; Field and Matheson, 1998). The localization of Para to the ciliary dilation is suggestive that Na_V_ channels may facilitate signal transduction at the ciliary dilation.

TRP channel opening typically occurs in response to mechanical, temperature, chemical, or noxious stimuli (Clapham et al., 2001; Montell et al., 2002; Zheng, 2013; Montell, 2021). However, many TRP channels have been shown to open in response to voltage (Hofmann et al., 2003; Nilius et al., 2003; Voets et al., 2004; Matta and Ahern, 2007). The rat TRP channels TRPV1 and TRPM8 are hot and cold responsive, respectively (Dhaka et al., 2006); however, under specific conditions, they can open in response to voltage (Voets et al., 2004; Matta and Ahern, 2007). At room temperature and physiological pH, TRPV1 opens at around 0 mV with a half-activation voltage of around 150 mV (Matta and Ahern, 2007). These activation ranges are a lot harder to achieve [compared with the properties of Na_V_1.2 (activation ∼−55 mV, half-activation voltage ∼−15 mV; Ogiwara et al., 2009)]. However, for these temperature-sensitive channels when the channel is exposed to higher temperatures the structure of the channel changes, and the half-activation threshold is far lower, −50 mV for TRPV1 at 42°C, which is readily achievable in a sensory neuron (Voets et al., 2004). It is not known whether the fly TRP channels are voltage sensitive, however, the two closest homologs of the vertebrate voltage sensing TRP channel *TRPV1* are *nan* (DIOPT 7/16) and *iav* (5/16; Hu et al., 2011). Interestingly, *iav* and *nan* are only expressed in the chordotonal PNS neuron dendrites where Para is localized (Kim et al., 2003; Gong et al., 2004). The possible voltage sensitivity of Iav/Nan in the same location as a Na_V_ channel suggests that Para activation could influence Iav/Nan activation or vice versa.

The dendrite of CNs is exposed to stretch forces opening mechanosensitive TRP channels. The presence of Para in the CN dendrite imposes the question as to whether Para may also be mechanosensitive. Two vertebrate Na_V_ channels Na_V_1.4 (*SCN4A*) and Na_V_1.5 (*SCN5A*) that are expressed in contracting muscle tissue have accelerated activation and inactivation kinetics when stretched in *Xenopus* oocytes (Shcherbatko et al., 1999; Tabarean et al., 1999; Morris and Juranka, 2007). While *in vivo* evidence for these channels’ mechanosensitivity is lacking, the G615E mutation in *SCN5A* which predominantly causes long-QT syndrome in patients has normal voltage-gating but aberrant mechanosensitivity indicating a role for Nav1.5 in mechanosensitivity (Strege et al., 2019). While *para* shares homology with *SCN4A* (DIOPT 9/16) and *SCN5A* (DIOPT 11/16; Hu et al., 2011), *para* is not expressed in muscle cells, and evidence for neuronal Na_V_ channel mechanosensitivity is less obvious (Morris, 2011). *para* has 60 isoforms with different protein sequences and different dynamic properties that likely accommodate the requirements of different neurons (Lin et al., 2009, 2012). An isoform-by-isoform expression study is needed to determine whether the CN *para* isoforms are closer in homology to *SCN5A* and *SCN4A* and thus more likely to be mechanosensitive.

In vertebrate neurons, voltage-gated ion channels can also contribute to graded potentials (Stuart et al., 1997; Golding and Spruston, 1998; Losonczy and Magee, 2006). In cultured hippocampal CA1 pyramidal neurons and brain slices of rat neocortical pyramidal neurons, spikes in membrane potential are observed in dendrites (Stuart et al., 1997; Losonczy and Magee, 2006; Sun et al., 2014). These dendritic spikes are not affected by the Ca_V_ channel blocker cadmium but are blocked by the Na_V_ channel blocker TTX, indicating that they are also generated by Na_V_ channels (Stuart et al., 1997). In CA1 pyramidal neurons, Na_V_1.6 is found in the dendrites, but it is 40 times less abundant than it is in axons (Lorincz and Nusser, 2010), while in the dendrites of *Drosophila* CNs, the max intensity of Para is roughly half that seen in the axon (Fig. 4*D*). Hence, Na_V_-dependent dendritic spikes are not an insect-specific phenomenon.

The inner hair cells of humans have a comparable organization to that of the fly CNs (Boekhoff-Falk, 2005). Studies in flies have elucidated key genes and mechanisms of the auditory response in humans (T. Li et al., 2018). One Na_V_ channel in vertebrates, *SCN8A*, has been implicated in a mouse model of peripheral hearing loss (Mackenzie et al., 2009). Interestingly, dominant variants in *SCN8A* are often implicated with more global neurodevelopmental disorders (Trudeau, 2006; Veeramah et al., 2012). The specific hearing loss identified in the prior mouse studies indicates a specific role in hearing for the affected residue. Na_V_ localization to the CNs in flies opens the door for the use of *Drosophila* to study the impact of Na_V_ dysfunction on hearing loss.

While we propose a role for Na_V_ channels in CN dendrites, the presence of Para in the AIS-like region in addition to the distal dendrites makes the delineation of Para’s role in either region impossible with current tools. Reduction of *para* expression using RNAi in the adult JO neurons shows a strong reduction of SEP indicating an inability to process sound. However, we are reducing Para in both the axons and the dendrites and therefore we cannot distinguish whether the loss of Para in dendrites prevents the dendritic depolarization from reaching the soma or whether the axonal AP is lost because of a lack of Para in the AIS. We tried to model dendritic CN activity in lch5 neurons using GCaMP7 (Dana et al., 2019) and TTX (Pauron et al., 1985) to block sodium channels but were unable to get reliable NaV channel inhibition, likely because of the insulating glial sheath, and the dynamics of the signal were too fast for reliable separation between axon and dendritic signal. To answer this question new tools are needed to selectively remove proteins in a compartment-specific way.

Para is localized to an AIS-like region in the axons of *Drosophila* PNS neurons. The region of Para localization overlaps with the previously reported AIS-like region in multipolar ddaE neurons, identified by an accumulation of over-expressed Ank2, Shal, and Elk and a diffusion barrier akin to the one seen at the vertebrate AIS (Jegla et al., 2016). The localization of Para and the AnkG homolog Ank2 in the AIS-like region of the PNS is of note as the AnkG binding motif in Na_V_ channels is not present in *para* indicating an alternative binding site and/or clustering mechanism in the fly PNS AIS-like region (Jegla et al., 2016).

In this study, we have identified the likely SIZ(s) in the multipolar and bipolar neurons of the fly PNS through the characterization of Na_V_ channel distribution. We have confirmed the presence of an axonal SIZ at an AIS-like region and surprisingly identified a likely dendritic SIZ in CNs throughout *Drosophila* development. The presence of Na_V_ channels in a dendritic and axonal SIZ in the fly PNS introduces an accessible system for further study into the role of Na_V_ channels in how animals sense their environment.

## Synthesis

Reviewing Editor: Miriam Goodman, Stanford University Decisions are customarily a result of the Reviewing Editor and the peer reviewers coming together and discussing their recommendations until a consensus is reached. When revisions are invited, a fact-based synthesis statement explaining their decision and outlining what is needed to prepare a revision will be listed below. The following reviewer(s) agreed to reveal their identity: Yu-Chieh Chen, Andrew Jarman. Note: If this manuscript was transferred from JNeurosci and a decision was made to accept the manuscript without peer review, a brief statement to this effect will instead be what is listed below.

This study makes use of reagents reported by this group in 2020 enabling the localization of endogenously tagged Para NaV channels in vivo. In this study, the authors show that the Para protein localizes to axons and dendrites. In chordotonal mechanosensory neurons, Para localizes to the distal sensory cilium near the zone that also contains the mechanosensitive ion channel, NOMPC. Reducing Para expressing impairs sound evoked potentials. From these findings, the authors indulge in quite a bit of speculation about the contribution of Para to sound responses in chordotonal organs, including a proposal that Para functions as an additional mechanosensitive protein in these sensory neurons. While this is possible, there is no functional evidence is available to distinguish between this possibility or the more classical idea that NaV channels provide positive feedback and amplify responses to depolarizing stimuli. In this context, the authors would be wise to alert readers to the speculative nature of these ideas and to discuss the type of studies that would be needed to distinguish between these possibilities.

Rev1 notes some concerns regarding terminology and figure composition. Addressing these comments will improve accessibility of this study to readers who are not already familiar with the Drosophila larval nervous system. Rev2 raises questions regarding anatomical assignments of dendritic compartments and asks for specificity of the antibody used to visualize the GFP tag. If this control was established previously, please provide a citation for these control experiments. If not, please perform experiments to determine antibody specificity (by labeling lines that lack the tag, for example).

Rev 1

The Transient Receptor Potential (TRP) channels in the Peripheral Nervous System (PNS) of the fruit fly Drosophila melanogaster have been studied to gain insights into sensory information transduction. However, these channels alone have not been sufficient to fully model mechanosensitive transduction in mechanoreceptive chordotonal neurons (CN). Following on their previous study of the voltage-gated sodium channel (NaV) Para in fly CNS, the authors used the same para reporter/protein trap, immunofluorescence imagining and electrophysiology recording to examine its expression in PNS from embryo to adult. The authors showed that Para is located at the distal tip of dendrites in all CNs, from embryos to adults, and is colocalized with mechanosensitive TRP channels NompC and Iav/Nan. The presence of Para also indicates spike initiation zones (SIZ) in axons, and its dendritic localization suggests a likely dendritic SIZ in fly CNs. Para knockdown in CNs in adult Johnston’s organ significantly impacts sound-evoked potentials.

In general, the characterization of para in Drosophila PNS provides an important entry point for further characterize the functional roles of para in different subcellular compartment, which will gain a better understanding of Para’s role in mechanosensitive transduction. Overall, the manuscript is clearly presented, and easy to follow. I do not have major concerns but have several minor suggestions outline below that could help the authors to improve the manuscript.

Minor concerns:

1. The authors mentioned in the text many times “multipolar”, “biopolar CN”, “embryonic CNs”, “multidendritic neurons” etc. It would be much more clear if the authors specified these different types of neurons in the introduction of at the beginning of the result section. Also, what features on the fluorescence staining were used to distinguish between these different PNS cell types?

2. Fig. 1b, box b, c, not box a? I find these boxes and ’, ’, ’, ’ quite distracting.

3. Some the terms have not yet defined, such as lCh5. SIZ should be defined again in the introduction.

4. The transition between embryonic/larval expression of para in CNs of the adult Johnston’s organ is quite abrupt. Is there any expression data of para in pupal Johnston’s organ neurons? These adult Johnston’s organ neurons are born during early pupation and the para expression during development would serve as a better transition into the functional role of para in mechanosensitive transduction.

Rev 2

While the overall message of this study is clear enough (intriguing expression of Nav in dendrites), there are some errors in interpretation of the chordotonal neuron localisations of Para protein that need to be corrected or at least justified.

P7 last paragraph and Fig. 1: para protein is localised to a “basket” beyond the tip of the dendrite. This does not seem to be consistent with a role within the dendrite itself and it is quite hard to visualise what structure this might correspond to as there is no clear analogous structure known from transmission electron microscopy of CNs.

P8 and Fig. 2: “In vpda neurons a dendrite can be seen in the proximal axon”. I’m not sure what this means or why it is mentioned. Have dendritic branches on the axon been identified in these neurons before?

P9 paragraph 1 and Fig. 3+4. The main Para localisation in the CN dendrites looks to me to be at the ciliary dilation in both figures, so that the last sentence of this paragraph is not accurate. The authors’ interpretation is based largely on the Eys marker, but Eys (Fig. 4A) is not a marker of the ciliary dilation in mature larval or JO chordotonal neurons (this is a misconception based on historical interpretation of embryonic expression patterns, see Styczynska-Soczka (2015) 10.1186/s13630-015-0018-9 for a counter example of the larval pattern). Therefore, the main expression is not beyond the CD. Colocalisation with a true CD marker would likely confirm that Para seems to be largely enriched at the ciliary dilation (and this would be a lot more consistent with the JO neuron expression shown later).

Fig. 4A: some apparent Para expression is noted distal to the dendritic cap, well beyond the dendrite. In our experience, GFP antibodies can produce artefacts such as this - have the authors conducted a control to check their antibodies on a non-tagged line?

P9 paragraph 2 and Fig. 5: in antennal neurons, expression is clearly in the region of the ciliary dilation. Such a localisation has interesting implications for Para’s role in the function of this enigmatic structure and this could be addressed more in the discussion?

Can the authors be reasonably certain that expression is in all JO neurons?

P9 “APs no longer detected”: it needs to be indicated in the figure what this refers to.

P10 paragraph 2: The comparison with the crayfish stretch receptor modelling is interesting and generally appropriate, but the authors mistakenly call these chordotonal neurons - they are not.

Discussion: this rather detailed, wide ranging and is obviously speculative at this point, but a good basis for future exploration of the interaction between para and TRP channels in mechanotransduction. The auditory phenotype clearly shows that para is required for CN function, but the interpretation is limited by the inability to pin this on axon or dendrite localisation. Perhaps the authors can suggest the use of Ca reporter imaging to obtain compartment-specific information?

## Author Response

Dear Dr. Goodman,

We appreciate the time spent by yourself and the reviewers in going over our manuscript and are grateful for the reviewer’s comments to ensure the manuscript is suitable for publication in eNeuro. Please find below the list of reviewers’ comments followed by our steps to address their concerns. Changes to the manuscript are in bold font to make them easy to identify.

Kind Regards

Hugo Bellen

Rev 1

The Transient Receptor Potential (TRP) channels in the Peripheral Nervous System (PNS) of the fruit fly Drosophila melanogaster have been studied to gain insights into sensory information transduction. However, these channels alone have not been sufficient to fully model mechanosensitive transduction in mechanoreceptive chordotonal neurons (CN). Following on their previous study of the voltage-gated sodium channel (NaV) Para in fly CNS, the authors used the same para reporter/protein trap, immunofluorescence imagining and electrophysiology recording to examine its expression in PNS from embryo to adult. The authors showed that Para is located at the distal tip of dendrites in all CNs, from embryos to adults, and is colocalized with mechanosensitive TRP channels NompC and Iav/Nan. The presence of Para also indicates spike initiation zones (SIZ) in axons, and its dendritic localization suggests a likely dendritic SIZ in fly CNs. Para knockdown in CNs in adult Johnston’s organ significantly impacts sound-evoked potentials.

In general, the characterization of para in Drosophila PNS provides an important entry point for further characterize the functional roles of para in different subcellular compartment, which will gain a better understanding of Para’s role in mechanosensitive transduction. Overall, the manuscript is clearly presented, and easy to follow. I do not have major concerns but have several minor suggestions outline below that could help the authors to improve the manuscript.

Minor concerns:

1. The authors mentioned in the text many times “multipolar”, “bipolar CN”, “embryonic CNs”, “multidendritic neurons” etc. It would be much more clear if the authors specified these different types of neurons in the introduction of at the beginning of the result section. Also, what features on the fluorescence staining were used to distinguish between these different PNS cell types?

2. Fig. 1b, box b, c, not box a? I find these boxes and ’, ’, ’, ’ quite distracting.

3. Some the terms have not yet defined, such as lCh5. SIZ should be defined again in the introduction.

4. The transition between embryonic/larval expression of para in CNs of the adult Johnston’s organ is quite abrupt. Is there any expression data of para in pupal Johnston’s organ neurons? These adult Johnston’s organ neurons are born during early pupation and the para expression during development would serve as a better transition into the functional role of para in mechanosensitive transduction.

We thank the reviewer for their kind comments and suggestions. To address these questions, we have made the following changes which are listed below corresponding to the number list the reviewer provided.

1. We added the following statement in the first paragraph of the results section to address the features we used to label the PNS cell types

a. “Multipolar neuron cell bodies have multiple dendritic processes with one axon, whereas bipolar neuron cell bodies have only one dendrite and one axon. These neurons can be easily identified by their location on the embryo (Singhania and Grueber, 2014): the dorsal cluster of neurons (Figure 1A, box ii) are all multipolar, a medial cluster of 5 neurons lined up parallel to each other are bipolar lateral chordotonal neurons (lch5) (Figure 1A, box i).”

2. We have addressed the confusion regarding the labeling of Figure one by separating it into two figures. Figure 1 is now just for gene expression in embryos and Figure 2 is for protein localization. This gave us room to annotate this figure in line with the rest of the manuscript.

3. We have added explanations for lch5 in the first paragraph of the results section and have included a description of the SIZ in the introduction.

a. “, a medial cluster of 5 neurons lined up parallel to each other are bipolar lateral chordotonal neurons (lch5)”

b. “In the unipolar neurons of the CNS, para is expressed in active, mature neurons and is localized to the spike initiation zone (SIZ), where action potentials are generated (AP),”

4. We thank the reviewer for their observation that the manuscript did not highlight that the dissections for Para localization and expression were done in pupae. We have added this explanation to the results section.

a. “The JO develops during pupal stages. Pupae 24-48h after puparium formation were dissected to observe Para localization and expression.”

Rev 2

While the overall message of this study is clear enough (intriguing expression of Nav in dendrites), there are some errors in interpretation of the chordotonal neuron localisations of Para protein that need to be corrected or at least justified.

P7 last paragraph and Fig. 1: para protein is localised to a “basket” beyond the tip of the dendrite. This does not seem to be consistent with a role within the dendrite itself and it is quite hard to visualise what structure this might correspond to as there is no clear analogous structure known from transmission electron microscopy of CNs.

We have removed the comparison of Para to a basket-like structure to avoid confusion and left the description as “Super-resolution stimulated emission depletion (STED) microscopy revealed that Para is present at the very distal tip of the dendrite with no membrane labeling via HRP present beyond where Para is localized (Figure 2C)” to describe that Para is present at the very tip of the dendrite.

P8 and Fig. 2: “In vpda neurons a dendrite can be seen in the proximal axon”. I’m not sure what this means or why it is mentioned. Have dendritic branches on the axon been identified in these neurons before?

These projections have been briefly reported by (Schrader and Merritt, 2000) to be present in some vpda neurons. They have not been studied or characterized extensively. We include this comment to describe that in situations where the dendritic branches of the axon are present, Para is distal to them, similar to what is seen in the central brain. “Additionally, in the vpda neuron, we observe dendrites that enter the axons beyond the cell body (Figure 3B). This branching has been previously reported to occur in some vpda neurons (Schrader and Merritt, 2000).”

P9 paragraph 1 and Fig. 3+4. The main Para localisation in the CN dendrites looks to me to be at the ciliary dilation in both figures, so that the last sentence of this paragraph is not accurate. The authors’ interpretation is based largely on the Eys marker, but Eys (Fig. 4A) is not a marker of the ciliary dilation in mature larval or JO chordotonal neurons (this is a misconception based on historical interpretation of embryonic expression patterns, see Styczynska-Soczka (2015) 10.1186/s13630-015-0018-9 for a counter-example of the larval pattern). Therefore, the main expression is not beyond the CD. Colocalization with a true CD marker would likely confirm that Para seems to be largely enriched at the ciliary dilation (and this would be a lot more consistent with the JO neuron expression shown later).

We have altered the description of Para and NompC localization to accurately reflect the chordotonal dendrite structure as highlighted by the reviewer. “NompC is localized at the ciliary dilation and at the tip of the dendrite, while Iav and Nan are localized proximal to the ciliary dilation (Figure 5E) (Gong, 2004; Lee et al., 2010; Liang et al., 2011). Para predominantly colocalizes with NompC at the tip of the dendrite (Figure 5C). Para also partially colocalizes with the more proximal Iav (Figure 5D), however, the majority of Para in dendrites is at the ciliary dilation together with NompC.” We also made changes to the respective figures to highlight this.

Fig. 4A: some apparent Para expression is noted distal to the dendritic cap, well beyond the dendrite. In our experience, GFP antibodies can produce artifacts such as this - have the authors conducted a control to check their antibodies on a non-tagged line?

To address this concern we have included a new supplemental figure, supplementary figure 4-1 that shows that the dendrite labeling is specific to the Para-GFP flies and is not present in wild-type animals.

P9 paragraph 2 and Fig. 5: in antennal neurons, expression is clearly in the region of the ciliary dilation. Such localization has interesting implications for Para’s role in the function of this enigmatic structure and this could be addressed more in the discussion?

We have edited the description in the results to emphasize the ciliary dilation localization “In these CNs, Para is also enriched in the dendrite, predominantly at the ciliary dilation (Figure 6B, 6C, Supplementary Video 1).” Additionally, we have added a paragraph in the discussion to briefly highlight para in the ciliary dilation and speculate that it may be responsible for the proposed signal transduction role of this structure “In lch5 and JO CNs Para is enriched at the ciliary dilation. The ciliary dilation is visible via a bulge in the membrane and is necessary for the separation of the dendrite into distinct regions (Lee et al. 2008). Beyond the ciliary dilation’s role in cellular organization, a role in signal transduction has been proposed but how this structure facilitates signal transduction is not clear (Moran D. et al 1977, Field L. and Matherson T. 1998). The localization of Para to the ciliary dilation is suggestive that NaV’s channels may facilitate signal transduction at the ciliary dilation.”

Can the authors be reasonably certain that expression is in all JO neurons?

We don’t see any of the F-actin-labeled scolopidia not connected to para-T2A-GAL4>UAS-mCD8::GFP labeled cells indicating that it is present in all. Additionally, single cell expression of the whole adult fly (Li H. et al, 2022) indicates all JO neurons express para. We have added this clarification to the manuscript “We do not observe any scolopodia, as labelled by F-Actin, that are not connected to a para expressing cell, therefore, like the CN in larvae and embryos, para is expressed in all the CNs of the JO (Figure 6A). This is consistent with the FlyCellAtlas which shows para expression in all JO neurons (Li et al. 2022).”

We have added some clarification on whether all cells in the JO have dendritic localization however as each scolopodia only has one para-enriched dendrite indicating some variation in the role of para in different JO neurons. We explain this in the results of the manuscript “Each scolopodium contains two or three JO neurons that are encased by the F-Actin spindles (Todi et al., 2004). Para is also enriched at the ciliary dilation of the dendrite in JO neurons, however in each scolopidium only one Para containing dendrite is observed (Figure 6B, 6C, Supplementary Video 1). Each scolopodium contains a stereotyped combination of neurons for detecting sound, wind and gravity respectively (Ishikawa et al., 2019). The restriction of Para localization to just one neuron’s dendrite indicates a different physiological role of NaV channels in different JO neuron types.” We added the supplemental video to show this clearer than it can be shown in a single image.

P9 “APs no longer detected”: it needs to be indicated in the figure what this refers to.

We have added in the text to reflect the description in the figure legend that the SEPs are strongly reduced as this is easier to see than describing a loss of AP “Additionally, when para expression is reduced, the SEPs in response to pulse stimulation are strongly reduced (Figure 6D, 6E).” Additionally, we included that the pulse song is represented by the pulse stimulus line in the figure legend “Acoustic near-field presentation of computer-generated pulse song stimulus (red pulse stim line) evokes strong SEPs in control animals with no Gal-4 driver.”

P10 paragraph 2: The comparison with the crayfish stretch receptor modelling is interesting and generally appropriate, but the authors mistakenly call these chordotonal neurons - they are not.

We have changed the naming of the crayfish stretch receptors “We believe this is a dendritic SIZ in agreement with a computational model of the crayfish stretch receptors for which NaV activation is needed to accurately represent in vivo recordings (Suslak et al., 2011).”

Discussion: this rather detailed, wide ranging and is obviously speculative at this point, but a good basis for future exploration of the interaction between para and TRP channels in mechanotransduction. The auditory phenotype clearly shows that para is required for CN function, but the interpretation is limited by the inability to pin this on axon or dendrite localization. Perhaps the authors can suggest the use of Ca reporter imaging to obtain compartment-specific information?

We tried to use GCaMP7 imaging in lch5 neurons while trying to block the channels with TTX. Two problems arose: getting reliable inhibition to the neuron wasn’t possible, likely due to the insulating glial sheath, and the reporter’s dynamics could not readily distinguish the dendritic from the axonal signal. We include a short segment into the discussion to address this obstacle we faced “We tried to model dendritic CN activity in lch5 neurons using GCaMP7 and TTX to block sodium channels but were unable to get reliable NaV channel inhibition, likely due to the insulating glial sheath, and the dynamics of the signal were too fast for reliable separation between axon and dendritic signal. To answer this question new tools are needed to selectively remove proteins in a compartment-specific way.”
